# Dietary Pattern of Garlic and Risk of Chronic Diseases: Evidence From Three Large‐Scale Cohorts

**DOI:** 10.1002/hcs2.70030

**Published:** 2025-08-03

**Authors:** Xiaoyu Tai, Tao Luo, Keying Song, Hui Zhao, Silu Chen, Huiqin Li, Min Liu, Jianghong Dai, Xu Qian, Mulong Du

**Affiliations:** ^1^ Department of Nutrition and Food Hygiene, Center for Global Health, School of Public Health Nanjing Medical University Nanjing China; ^2^ Department of Epidemiology and Biostatistics, School of Public Health Xinjiang Medical University Urumqi China; ^3^ Department of Environmental Genomics and Genetic Toxicology, The Key Laboratory of Modern Toxicology of the Ministry of Education, Center for Global Health, Jiangsu Key Laboratory of Cancer Biomarkers, Prevention and Treatment, Collaborative Innovation Center for Cancer Personalized Medicine, School of Public Health Nanjing Medical University Nanjing China; ^4^ Jiangsu Cancer Hospital, Nanjing Medical University Affiliated Cancer Hospital, Jiangsu Institute of Cancer Research Nanjing China; ^5^ Jiangsu Key Lab of Cancer Biomarkers, Prevention and Treatment, Collaborative Innovation Center for Cancer Personalized Medicine Nanjing Medical University Nanjing China; ^6^ Department of Biostatistics, Center for Global Health, School of Public Health, and Department of Urology The Second Affiliated Hospital of Nanjing Medical University, Nanjing Medical University Nanjing China

**Keywords:** chronic disease, cohort study, garlic

## Abstract

**Background:**

Understanding the relationship between garlic and chronic diseases could help to improve prevention and reduce the burden of diseases. This study aimed to examine the association between garlic consumption and the risk of chronic diseases.

**Methods:**

We included 26,524 participants from the Chinese Longitudinal Healthy Longevity Survey (CLHLS) with data of the frequency of garlic consumption, 7658 participants from the Xinjiang multiethnic cohort (XMC) study with data of garlic intake, and 141,684 participants from the UK Biobank (UKBB) with data of the preference for garlic. The dietary pattern of garlic, including the frequency of consuming garlic, garlic intake, and garlic preference information, was collected using a food questionnaire for each cohort. Logistic regression and structural equation modeling were used to assess the effect of garlic consumption on five common chronic diseases, which comprised cancer, diabetes, hypertension, respiratory diseases, and cardiovascular disease (CVD).

**Results:**

In the CLHLS cohort, individuals who consumed garlic almost every day had a significantly lower risk of five of the most common chronic diseases (cancer: odds ratio [OR] = 0.51, 95% confidence interval [CI] = 0.30–0.81, *p* = 0.006; diabetes: OR = 0.58, 95% CI = 0.43–0.76, *p* < 0.001; hypertension: OR = 0.68, 95% CI = 0.61–0.77, *p* < 0.001; respiratory diseases: OR = 0.77, 95% CI = 0.67–0.87, *p* < 0.001; and CVD: OR = 0.69, 95% CI = 0.59–0.80, *p* < 0.001). Similarly, in the XMC, there was a consistent protective effect of high garlic intake on hypertension, respiratory diseases and CVD. Additionally, in the UKBB cohort, individuals who liked garlic had a decreased risk of diabetes and CVD. Notably, in three cohorts, structural equation modeling results showed that there was a significant protective total effect of garlic consumption on the five common chronic diseases.

**Conclusions:**

A high garlic consumption is associated with a reduced risk of chronic diseases. Our findings highlight the potential protective role of garlic in preventing chronic diseases.

## Graphical abstract

Upon three large‐scale cohorts, we investigated three dietary patterns of garlic (i.e., garlic frequency, garlic intake, and garlic preference) on five common chronic diseases risk, and observed a protective effect of high garlic consumption on reducing risk of chronic diseases.

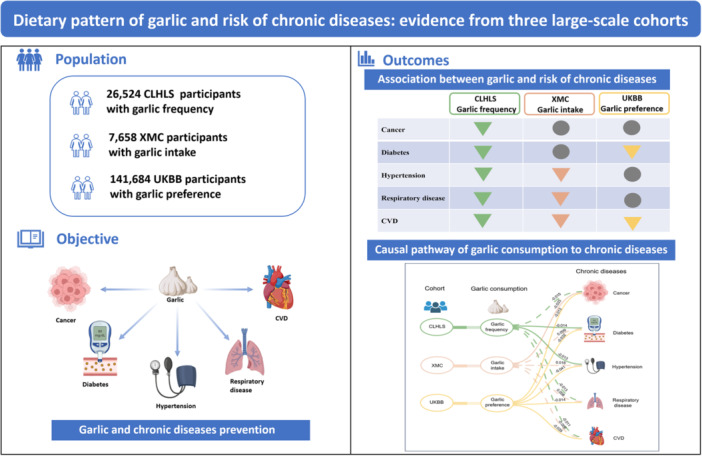

**Abbreviations**
BMI: body mass index
CI: confidence interval
CLHLS: Chinese Longitudinal Healthy Longevity Survey
CVD: cardiovascular disease
OR: odds ratio
SEM: structural equation modeling
UKBB: UK Biobank
XMC: Xinjiang multiethnic cohort

## 1 Introduction

Garlic (*Allium sativum* L.) is a common allium plant that is widely used worldwide as a flavoring and medicinal herb. Garlic contains a variety of phytochemicals, such as organosulfur compounds, phenolic compounds, saponins, and polysaccharides [1, 2, 3]. In particular, allicin is an important sulfur‐containing compound in garlic that contributes to its unique odor. These active ingredients interact with each other and provide beneficial health effects [4].

Numerous animal experiments and clinical trials have demonstrated that garlic possesses a range of biological functions, such as anticancer, antidiabetic, antihypertensive, and antibacterial effects [5, 6, 7]. Additionally, several cohort studies have reported that habitual garlic consumption is associated with a lower risk of all‐cause mortality and the development of cancer [8, 9]. A longitudinal follow‐up study in Iran showed that habitual intake of allium vegetables was associated with a decreased risk of cardiovascular disease (CVD), hypertension, and chronic kidney disease, but did not show a significant association with the risk of diabetes [10]. However, two cohort studies in the USA failed to establish a relationship between a high garlic intake and a lower risk of colorectal cancer [11]. Most of these studies of garlic focused on a single dietary pattern, and did not comprehensively evaluate the health effects of diverse garlic dietary patterns (i.e., frequency, intake, and preference).

Dietary behavior is a multifaceted process that encompasses not only the frequency and quantity of food intake, but also cultural influences, individual preferences, and interactions between different foods [12]. These factors can effect health outcomes by affecting the release and metabolism of active ingredients. Therefore, the approach of using detailed cohort data to consider different garlic dietary patterns could more accurately reflect the effect of individual dietary habits on health and provide a more scientific basis for developing precise dietary guidelines.

In this study, we used logistic regression models and structural equation modeling (SEM) to assess the relationship between garlic consumption (i.e., frequency of garlic consumption, garlic intake, and preference for garlic) and the risk of five of the most common chronic diseases (i.e., cancer, diabetes, hypertension, respiratory diseases, and CVD) using data from three large‐scale cohorts: the Chinese Longitudinal Healthy Longevity Survey (CLHLS), the Xinjiang multiethnic cohort (XMC), and the UK Biobank (UKBB).

## 2 Materials and Methods

### 2.1 Study Population

The CLHLS was a prospective cohort study that began in 1998, and focused on older adults aged 65 years and older. It covered 23 provinces and regions of China. Face‐to‐face interviews were used to collect data on demographic characteristics, basic family status, economic status, ability to perform daily activities, and disease status of the participants. A more comprehensive description of the CLHLS has been published elsewhere [13, 14]. This study enrolled 33,213 participants from 1998 to 2011. After excluding individuals with abnormal baseline data (*n* = 6192), we included 27,021 participants from this cohort.

The XMC study is a large‐scale prospective cohort study that is currently ongoing in Xinjiang, China. The XMC study has recruited 30,949 individuals aged 35–74 years from three sites—Urumqi, Hotan Prefecture, and Ili Kazakh Autonomous Prefecture, since 2018. The XMC study collected demographics, dietary and lifestyle factors, and other relevant information by face‐to‐face questionnaires, physical examinations, and biospecimen collection. The cohort profile has been published elsewhere [15]. In this study, we included only 8011 participants from the Ili region because of the availability of detailed dietary data, specifically regarding garlic intake, which was only investigated in that region. A semi‐quantitative food frequency questionnaire was administered only to participants in the Ili region. Individuals without completed dietary questionnaires (*n* = 252) or with duplicate IDs (*n* = 51) were excluded.

The UKBB study is a large‐scale, prospective study that recruited more than 500,000 participants aged between 40 and 69 years from 22 assessment centers located across England, Wales, and Scotland from 2006 to 2010. Design and methodology information has been described elsewhere in more detail [16]. Quality control of populations was followed in accordance with our previously published studies [17]: (1) we excluded individuals of gender discordance; (2) we excluded outliers with genotypic deletions or excessive heterozygosity; (3) we retained unrelated participants; (4) we excluded “others” and retained only “White British” participants; and (5) we excluded individuals who decided not to participate in this project. We finally included 378,487 participants. The UKBB data are based on application number #45611.

**Figure 1:**
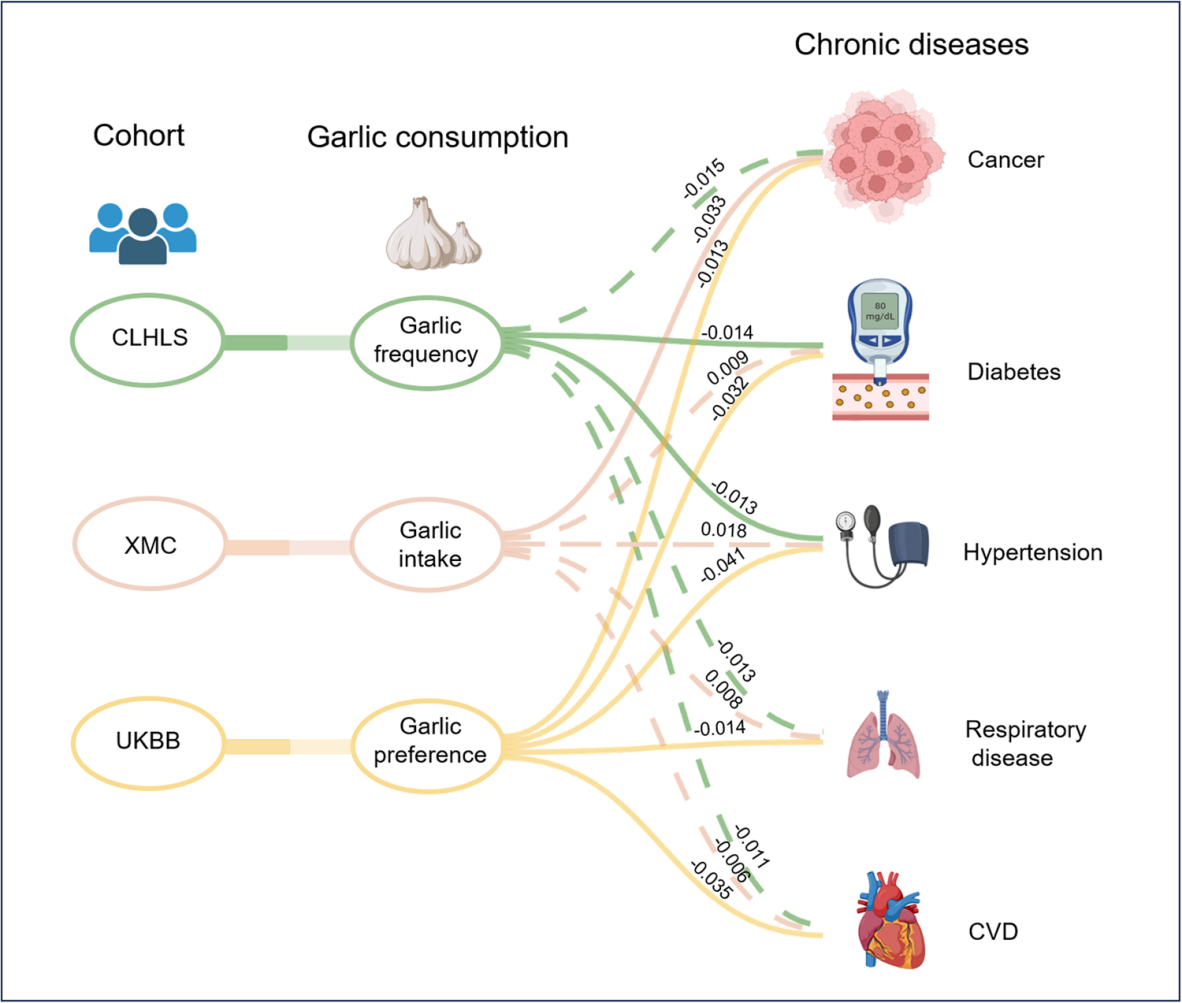
Total effects of garlic consumption and chronic diseases by structural equation modelling for the CLHLS, XMC and UKBB cohorts. CLHLS, Chinese Longitudinal Healthy Longevity Survey; CVD, cardiovascular disease; UKBB, UK Biobank; XMC, Xinjiang multiethnic cohort.

In all three cohorts, we excluded individuals without information on garlic and those with one or more of the five common chronic diseases at baseline. Overall, 26,524 participants in the CLHLS, 7658 participants in the XMC study, and 141,684 participants in the UKBB study were included in this study (Supporting Information S1: Figure 1).

### 2.2 Assessment of Garlic Dietary Patterns

#### 2.2.1 Frequency of Consuming Garlic

In the CLHLS baseline and follow‐up questionnaires, the participants were asked “How often do you eat garlic at present?” The participants provided responses such as “almost every day,” “occasionally,” or “rarely or never.” In this study, we categorized the frequency of eating garlic into three groups on the basis of responses from the baseline and follow‐up questionnaires: “rarely or never,” “occasionally,” and “almost every day.”

#### 2.2.2 Garlic Intake

In the dietary questionnaire for the XMC, the participants were asked “How often did you consume garlic in the past year?” The participants provided responses indicating the frequency and amount of garlic intake for each serving. To determine the average weekly garlic intake (g/week), we multiplied the frequency of garlic intake (times/week) by the amount per intake (g). Subsequently, the participants were categorized into quartiles on the basis of their weekly garlic intake. In the CLHLS cohort and XMC, information on garlic was collected using a Food Frequency Questionnaire, which has been validated for reliability and accuracy in previous studies [18, 19].

#### 2.2.3 Preference for Garlic

In the online questionnaire of the UKBB cohort, food‐liking traits were collected. The participants rated their liking for garlic on a scale of 1 (extremely dislike) to 9 (extremely like) to indicate their preference level. We further defined a preference for garlic as “dislike” (scores of 1–4), “neither like nor dislike” (score of 5), and “like” (scores of 6–9). The UKBB food preference questionnaire is an extension of those used by Pallister et al. and Vink et al., and it has demonstrated good statistical properties [20, 21].

### 2.3 Ascertainment of Chronic Diseases

In this study, we investigated five common chronic diseases, namely cancer, diabetes, hypertension, respiratory diseases, and CVD [22, 23]. In the CLHLS cohort and XMC, we identified the presence of chronic diseases on the basis of a self‐reported physician diagnosis of medical conditions. In the UKBB cohort, we used the International Classification of Diseases, 10th Revision [17, 24, 25] to identify participants with chronic diseases (Supporting Information S2: Table 1).

### 2.4 Selection of Covariates

Covariate information of the three cohorts was collected through questionnaires. Physical characteristics included age, sex, and ethnicity (CLHLS and XMC). Lifestyle factors included smoking status, drinking status, exercise, self‐reported health, and body mass index (BMI). Furthermore, dietary factors included consumption of fruit, vegetables, meat, fish, eggs, beans, sweets, tea, and salt‐preserved vegetables, as well as dietary patterns. More details regarding the covariates are shown in the Supporting Information.

### 2.5 Statistical Analysis

We described baseline characteristics based on information on garlic from the three cohorts, with continuous variables expressed as the mean (standard deviation) and categorical variables expressed as the number (%). Logistic regression analyses were used to estimate the odds ratio (OR) and 95% confidence interval (CI) between garlic and the risk of chronic diseases. We constructed a crude model and three multivariate models as follows. Model 1 included age, sex, smoking status, and drinking status. Model 2 included the variables in Model 1 and was adjusted for additional exercise, self‐reported health, BMI, and dietary factors (e.g., consumption of fruit, vegetables, meat, fish, eggs, beans, sweets, tea, and salt‐preserved vegetables). Model 3 included the variables in Model 1 and was adjusted for additional exercise, self‐reported health, BMI, and dietary patterns. Finally, we used SEM to examine the direct effects of garlic on the risk of chronic diseases while considering the indirect effects of garlic on the risk of chronic diseases through physical, lifestyle, and dietary factors. The total effect was the sum of the direct and indirect effects, and was calculated by the following equation: c = c′ + ab, where c = total effect, c′ = direct effect, and ab = indirect effect [26]. Structural Equation Modeling (SEM) was run by AMOS 24.0 (International Business Machines Corporation, America) and all other statistical analyses were analyzed using R V. 4.2.1 (R Foundation, New Zealand).

## 3 Results

### 3.1 Baseline Characteristics of the Cohorts

Supporting Information S2: Table 2 shows the baseline characteristics of the study participants from the CLHLS cohort, XMC, and UKBB cohort according to their garlic dietary pattern. We included 26,524 participants in the CLHLS cohort, 7658 participants in the XMC, and 141,684 participants in the UKBB cohort for further association analyses.

### 3.2 Associations Between Garlic and the Risk of Chronic Diseases

After adjusting for demographics, lifestyle, and dietary patterns, garlic consumption was consistently associated with a reduced risk of the five common chronic diseases (Table 1). In the CLHLS cohort, participants who consumed garlic almost every day had a 49% lower risk of cancer (OR = 0.51, 95% CI = 0.30–0.81, *p* = 0.006), a 42% lower risk of diabetes (OR = 0.58, 95% CI = 0.43–0.76, *p* < 0.001), a 32% lower risk of hypertension (OR = 0.68, 95% CI = 0.61–0.77, *p* < 0.001), a 23% lower risk of respiratory diseases (OR = 0.77, 95% CI = 0.67–0.87, *p* < 0.001), and a 31% lower risk of CVD (OR = 0.69, 95% CI = 0.59–0.80, *p* < 0.001) compared with those who rarely or never consumed garlic (Table 2). Similar findings were observed in the XMC and UKBB cohort. Regarding weekly garlic intake of participants in the XMC (Table 3), the second quartile of garlic intake was associated with a 25% reduced risk of hypertension (OR = 0.75, 95% CI = 0.62–0.90, *p* = 0.003), a 32% reduced risk of respiratory diseases (OR = 0.68, 95% CI = 0.53–0.94, *p* = 0.017), and a 49% reduced risk of CVD (OR = 0.51, 95% CI = 0.30–0.86, *p* = 0.011), using the lowest quartile group as a reference. Regarding the preference of garlic in the UKBB cohort, the protective effect of liking garlic showed a 20% decreased risk of diabetes (OR = 0.80, 95% CI = 0.73–0.88, *p* < 0.001) and a 9% decreased risk of CVD (OR = 0.91, 95% CI = 0.85–0.98, *p* < 0.009) compared with a dislike of garlic (Table 4).

**Table 1:** Associations between garlic and the risk of chronic diseases in the CLHLS cohort, XMC, and UKBB cohort using Model 3.

	CLHLS					XMC					UKBB				
	Garlic frequency	Total	Cases	OR (95% CI)	*p*	Garlic intake	Total	Cases	OR (95% CI)	*p*	Garlic preference	Total	Cases	OR(95% CI)	*p*
Model3^a^															
Cancer						Quartile 1	2146	18	Reference						
	Rarely or never	13,212	100	Reference		Quartile 2	2869	9	0.45 (0.18–1.06)	0.072	Dislike	17,054	1131	Reference	
	Occasionally	9335	43	0.59 (0.40–0.86)	0.007	Quartile 3	1385	4	0.41 (0.11–1.23)	0.138	Neither like nor dislike	10,012	605	0.94 (0.84–1.04)	0.232
	Almost everyday	3977	21	0.51 (0.30–0.81)	0.006	Quartile 4	1258	1	0.14 (0.02–1.10)	0.062	Like	114,618	6564	1.03 (0.96–1.11)	0.357
Diabetes						Quartile 1	2146	103	Reference						
	Rarely or never	13,212	268	Reference		Quartile 2	2869	110	0.81 (0.59–1.11)	0.189	Dislike	17,054	792	Reference	
	Occasionally	9335	145	0.69 (0.55–0.86)	< 0.001	Quartile 3	1385	66	1.04 (0.72–1.51)	0.821	Neither like nor dislike	10,012	466	1.00 (0.88–1.14)	0.965
	Almost everyday	3977	64	0.58 (0.43–0.76)	< 0.001	Quartile 4	1258	69	1.26 (0.86–1.83)	0.236	Like	114,618	3514	0.80 (0.73–0.88)	< 0.001
Hypertension						Quartile 1	2146	378	Reference						
	Rarely or never	13,212	1945	Reference		Quartile 2	2869	406	0.75 (0.62–0.90)	0.003	Dislike	17,054	3446	Reference	
	Occasionally	9335	1,201	0.77 (0.71–0.84)	< 0.001	Quartile 3	1385	260	1.17 (0.94–1.46)	0.156	Neither like nor dislike	10,012	1953	0.97 (0.91–1.05)	0.467
	Almost everyday	3977	559	0.68 (0.61–0.77)	< 0.001	Quartile 4	1258	225	1.14 (0.91–1.43)	0.245	Like	114,618	18,236	0.97 (0.93–1.02)	0.258
Respiratory diseases						Quartile 1	2146	108	Reference						
	Rarely or never	13,212	1235	Reference		Quartile 2	2869	96	0.68 (0.53–0.94)	0.017	Dislike	17,054	1355	Reference	
	Occasionally	9335	698	0.74 (0.67–0.82)	< 0.001	Quartile 3	1385	64	0.96 (0.67–1.38)	0.835	Neither like nor dislike	10,012	734	0.93 (0.84–1.03)	0.167
	Almost everyday	3977	355	0.77 (0.67–0.87)	< 0.001	Quartile 4	1258	63	1.14 (0.78–1.64)	0.493	Like	114,618	7911	0.95 (0.89–1.02)	0.131
CVD						Quartile 1	2146	42	Reference						
	Rarely or never	13,212	877	Reference		Quartile 2	2869	34	0.51 (0.30–0.86)	0.011	Dislike	17,054	1375	Reference	
	Occasionally	9335	473	0.69 (0.61–0.78)	< 0.001	Quartile 3	1385	23	0.73 (0.39–1.30)	0.289	Neither like nor dislike	10,012	749	0.94 (0.85–1.05)	0.286
	Almost everyday	3977	258	0.69 (0.59–0.80)	< 0.001	Quartile 4	1258	23	0.83 (0.44–1.51)	0.545	Like	114,618	6512	0.91 (0.85–0.98)	0.009

Abbreviations: 95% CI, 95% confidence interval; CLHLS, Chinese Longitudinal Healthy Longevity Survey; CVD, cardiovascular disease; OR, odds ratio; UKBB, UK Biobank; XMC, Xinjiang multiethnic cohort.

^a^ Adjusted for age, sex, smoking status, drinking status, body mass index, ethnicity (CLHLS, XMC), exercise, self‐reported health, and dietary pattern.

**Table 2:** Associations between the frequency of garlic consumption and the risk of chronic diseases in the CLHLS cohort.

	Garlic frequency	Total	Case	Crude		Model 1^a^		Model 2^b^		Model 3^c^	
				OR (95% CI)	*p*	OR (95% CI)	*p*	OR (95% CI)	*p*	OR (95% CI)	*p*
Cancer	Rarely or never	13,212	100	Reference		Reference		Reference		Reference	
	Occasionally	9335	43	0.72 (0.50–1.02)	0.071	0.64 (0.44–0.92)	0.017	0.55 (0.38–0.80)	0.002	0.59 (0.40–0.86)	0.007
	Almost everyday	3977	21	0.74 (0.45–1.16)	0.209	0.55 (0.33–0.87)	0.014	0.46 (0.27‐0.74)	0.002	0.51 (0.30–0.81)	0.006
Diabetes	Rarely or never	13,212	268	Reference		Reference		Reference		Reference	
	Occasionally	9335	145	0.78 (0.64–0.96)	0.018	0.72 (0.58–0.88)	0.002	0.69 (0.55–0.86)	< 0.001	0.69 (0.55–0.86)	< 0.001
	Almost everyday	3977	64	0.82 (0.62–1.07)	0.155	0.60 (0.45–0.79)	< 0.001	0.61 (0.45–0.82)	0.001	0.58 (0.43–0.76)	< 0.001
Hypertension	Rarely or never	13,212	1945	Reference		Reference		Reference		Reference	
	Occasionally	9335	1201	0.88 (0.81–0.95)	< 0.001	0.77 (0.71–0.84)	< 0.001	0.77 (0.70–0.84)	< 0.001	0.77 (0.71–0.84)	< 0.001
	Almost everyday	3977	559	0.98 (0.89–1.09)	0.747	0.70 (0.63–0.78)	< 0.001	0.75 (0.78–0.93)	< 0.001	0.68 (0.61–0.77)	< 0.001
Respiratory diseases	Rarely or never	13,212	1235	Reference		Reference		Reference		Reference	
	Occasionally	9335	698	0.79 (0.72–0.87)	< 0.001	0.73 (0.66–0.80)	< 0.001	0.74 (0.66–0.82)	< 0.001	0.74 (0.67–0.82)	< 0.001
	Almost everyday	3977	355	0.97 (0.85–1.09)	0.598	0.77 (0.68–0.87)	< 0.001	0.81 (0.70–0.92)	0.002	0.77 (0.67–0.87)	< 0.001
CVD	Rarely or never	13,212	877	Reference		Reference		Reference		Reference	
	Occasionally	9335	473	0.76 (0.67–0.85)	< 0.001	0.68 (0.60–0.77)	< 0.001	0.69 (0.61–0.78)	< 0.001	0.69 (0.61–0.78)	< 0.001
	Almost everyday	3977	258	0.98 (0.85–1.13)	0.763	0.71 (0.61–0.82)	< 0.001	0.72 (0.62–0.84)	< 0.001	0.69 (0.59–0.80)	< 0.001

Abbreviations: 95% CI, 95% confidence interval; CLHLS, Chinese Longitudinal Healthy Longevity Survey; CVD, cardiovascular disease; OR, odds ratio.

^a^ Model 1 was adjusted for age, sex, smoking status, and drinking status.

^b^ Model 2 was adjusted for age, sex, smoking status, drinking status, body mass index, ethnicity, exercise, self‐reported health, and dietary factors (consumption of fruit, vegetables, meat, fish, eggs, beans, sweets, tea, and salt‐preserved vegetables).

^c^ Model 3 was adjusted for age, sex, smoking status, drinking status, body mass index, ethnicity, exercise, self‐reported health, and dietary pattern.

**Table 3:** Associations between garlic intake and the risk of chronic diseases in the XMC.

	Garlic intake	Total	Cases	Crude		Model 1^a^		Model 2^b^		Model 3^c^	
				OR (95% CI)	*p*	OR (95% CI)	*p*	OR (95% CI)	*p*	OR (95% CI)	*p*
Cancer	Quartile 1	2146	18	Reference		Reference		Reference		Reference	
	Quartile 2	2869	9	0.37 (0.16–0.81)	0.016	0.41 (0.17–0.90)	0.031	0.49 (0.19–1.29)	0.149	0.45 (0.18–1.06)	0.072
	Quartile 3	1385	4	0.34 (0.12–1.01)	0.053	0.37 (0.12–1.09)	0.072	0.43 (0.11–1.37)	0.176	0.41 (0.11–1.23)	0.138
	Quartile 4	1258	1	0.09 (0.01–0.46)	0.021	0.10 (0.01–0.50)	0.027	0.13 (0.02–1.06)	0.056	0.14 (0.02–1.10)	0.062
Diabetes	Quartile 1	2146	103	Reference		Reference		Reference		Reference	
	Quartile 2	2869	110	0.79 (0.60–1.04)	0.094	0.83 (0.63–1.11)	0.210	0.92 (0.66–1.29)	0.636	0.81 (0.59–1.11)	0.189
	Quartile 3	1385	66	0.99 (0.72–1.36)	0.963	1.00 (0.72–1.38)	0.992	1.06 (0.72–1.55)	0.756	1.04 (0.72–1.51)	0.821
	Quartile 4	1258	69	1.15 (0.84–1.57)	0.379	1.14 (0.82–1.57)	0.440	1.32 (0.89–1.94)	0.163	1.26 (0.86–1.83)	0.236
Hypertension	Quartile 1	2146	378	Reference		Reference		Reference		Reference	
	Quartile 2	2869	406	0.77 (0.66–0.90)	< 0.001	0.75 (0.64–0.89)	< 0.001	0.78 (0.64–0.94)	0.011	0.75 (0.62–0.90)	0.003
	Quartile 3	1385	260	1.08 (0.91–1.29)	0.382	1.03 (0.86–1.25)	0.718	1.14 (0.91–1.42)	0.255	1.17 (0.94–1.46)	0.156
	Quartile 4	1258	225	1.02 (0.85–1.22)	0.841	0.92 (0.76–1.12)	0.400	1.13 (0.90–1.43)	0.285	1.14 (0.91–1.43)	0.245
Respiratory diseases	Quartile 1	2146	108	Reference		Reference		Reference		Reference	
	Quartile 2	2869	96	0.65 (0.49–0.86)	0.003	0.66 (0.50–0.88)	0.005	0.67 (0.48–0.93)	0.016	0.68 (0.53–0.94)	0.017
	Quartile 3	1385	64	0.91 (0.66–1.25)	0.579	0.90 (0.65–1.24)	0.526	0.89(0.61‐1.29)	0.537	0.96 (0.67–1.38)	0.835
	Quartile 4	1258	63	0.99 (0.72–1.36)	0.975	0.98 (0.70–1.35)	0.894	1.04 (0.71–1.52)	0.825	1.14 (0.78–1.64)	0.493
CVD	Quartile 1	2146	42	Reference		Reference		Reference		Reference	
	Quartile 2	2869	34	0.60 (0.38–0.95)	0.028	0.60 (0.38–0.95)	0.031	0.62 (0.36–1.07)	0.084	0.51 (0.30–0.86)	0.011
	Quartile 3	1385	23	0.85 (0.50–1.40)	0.523	0.81 (0.47–1.34)	0.412	0.76 (0.40–1.39)	0.373	0.73 (0.39–1.30)	0.289
	Quartile 4	1258	23	0.93 (0.55–1.54)	0.791	0.84 (0.49–1.41)	0.519	0.87 (0.45–1.62)	0.661	0.83 (0.44–1.51)	0.545

Abbreviations: 95% CI, 95% confidence interval; CVD, cardiovascular disease; OR, odds ratio; XMC, Xinjiang multiethnic cohort.

^a^ Model 1 was adjusted for age, sex, smoking status, and drinking status.

^b^ Model 2 was adjusted for age, sex, smoking status, drinking status, body mass index, ethnicity, exercise, self‐reported health, and dietary factors (consumption of fruit, vegetables, meat, fish, eggs, beans, sweets, tea, and salt‐preserved vegetables).

^c^ Model 3 was adjusted for age, sex, smoking status, drinking status, body mass index, ethnicity, exercise, self‐reported health, and dietary pattern.

**Table 4:** Associations between the preference for garlic and the risk of chronic diseases in the UKBB cohort.

	Garlic preference	Total	Case	Crude		Model 1^a^		Model 2^b^		Model 3^c^	
				OR (95% CI)	*p*	OR (95% CI)	*p*	OR (95% CI)	*p*	OR (95% CI)	*p*
Cancer	Dislike	17,054	1131	Reference		Reference		Reference		Reference	
	Neither like nor dislike	10,012	605	0.91 (0.82–1)	0.056	0.93 (0.84–1.03)	0.145	0.94 (0.84–1.04)	0.231	0.94 (0.84–1.04)	0.232
	Like	114,618	6564	0.86 (0.8–0.91)	< 0.001	1.00 (0.94–1.07)	0.910	1.03 (0.96–1.1)	0.481	1.03 (0.96–1.11)	0.357
Diabetes	Dislike	17,054	792	Reference		Reference		Reference		Reference	
	Neither like nor dislike	10,012	466	0.95 (0.89–1.02)	0.135	1.02 (0.91–1.15)	0.716	1.03 (0.90–1.17)	0.666	1.00 (0.88–1.14)	0.965
	Like	114,618	3514	0.74 (0.71–0.77)	< 0.001	0.74 (0.68–0.81)	< 0.001	0.85 (0.78–0.93)	< 0.001	0.80 (0.73–0.88)	< 0.001
Hypertension	Dislike	17,054	3446	Reference		Reference		Reference		Reference	
	Neither like nor dislike	10,012	1953	0.95 (0.89–1.01)	0.132	0.99 (0.93–1.06)	0.836	0.98 (0.92–1.05)	0.614	0.97 (0.91–1.05)	0.467
	Like	114,618	18,236	0.74 (0.71–0.77)	< 0.001	0.92 (0.88–0.96)	< 0.001	1 (0.96–1.05)	0.922	0.97 (0.93–1.02)	0.258
Respiratory diseases	Dislike	17,054	1355	Reference		Reference		Reference		Reference	
	Neither like nor dislike	10,012	734	0.90 (0.82–1.00)	0.042	0.91 (0.83–1.01)	0.073	0.93 (0.84–1.03)	0.173	0.93 (0.84–1.03)	0.167
	Like	114,618	7911	0.84 (0.79–0.90)	< 0.001	0.87 (0.82–0.93)	< 0.001	0.98 (0.91–1.04)	0.462	0.95 (0.89–1.02)	0.131
CVD	Dislike	17,054	1375	Reference		Reference		Reference		Reference	
	Neither like nor dislike	10,012	749	0.93 (0.84–1.02)	0.13	0.96 (0.87–1.06)	0.420	0.96 (0.87–1.06)	0.446	0.94 (0.85–1.05)	0.286
	Like	114,618	6512	0.68 (0.64–0.73)	< 0.001	0.85 (0.79–0.90)	< 0.001	0.94 (0.88–1.01)	0.076	0.91 (0.85–0.98)	0.009

Abbreviations: 95% CI, 95% confidence interval; CVD, cardiovascular disease; OR, odds ratio; UKBB, UK Biobank.

^a^ Model 1 was adjusted for age, sex, smoking status, and drinking status.

^b^ Model 2 was adjusted for age, sex, smoking status, drinking status, body mass index, exercise, self‐reported health, and dietary factors (consumption of fruit, vegetables, red meat, processed meat, fish, and tea), egg preferences, bean preferences, salty food preferences, and sweet food preferences.

^c^ Model 3 was adjusted for age, sex, smoking status, drinking status, body mass index, exercise, self‐reported health, and dietary pattern.

### 3.3 Causal Pathway of Garlic Consumption to Chronic Diseases

**Figure 2:**
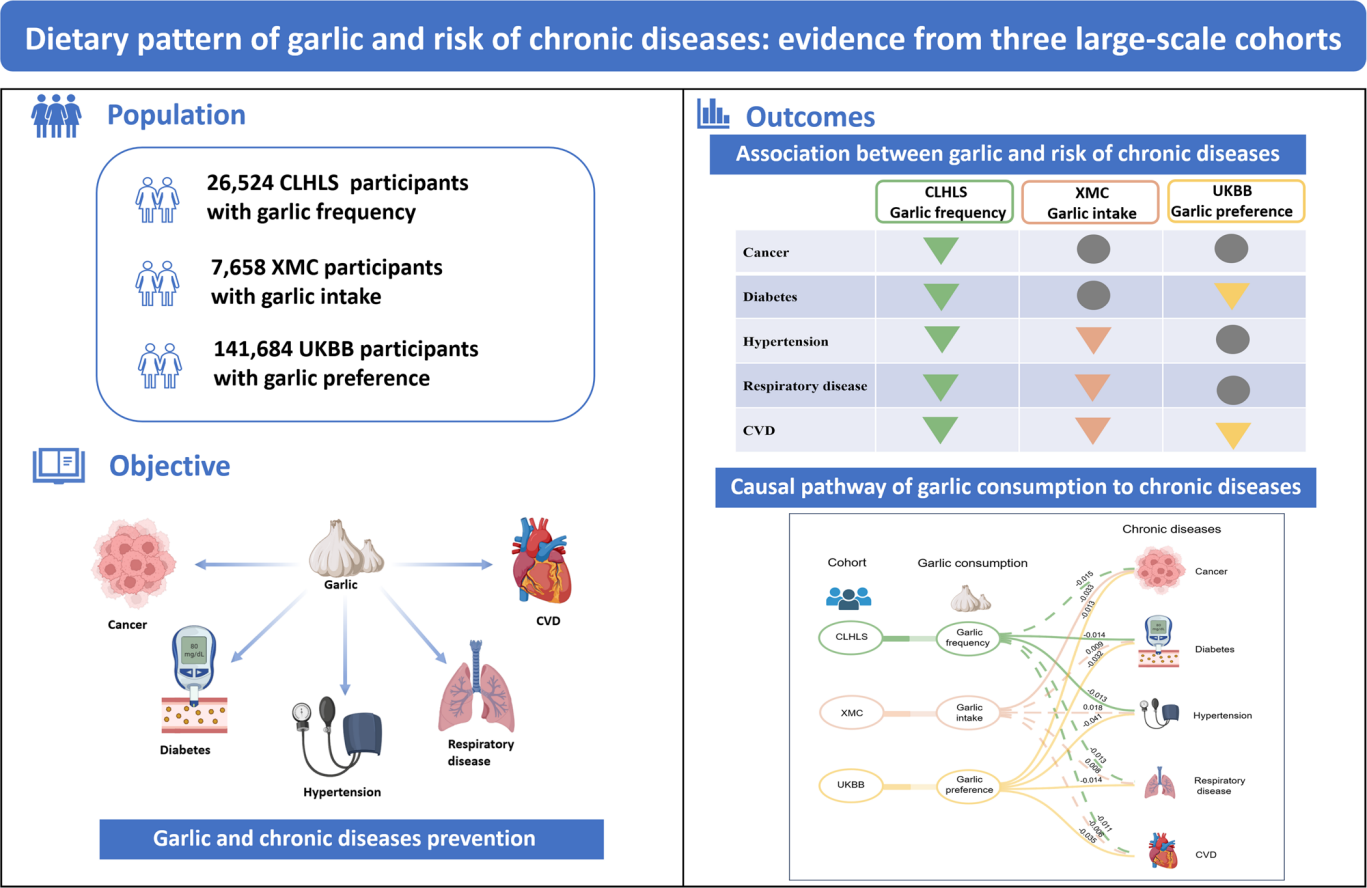
Garlic consumption of and chronic diseases risk: results from three cohort studies. CLHLS, Chinese Longitudinal Healthy Longevity Survey; CVD, cardiovascular disease; UKBB, UK Biobank; XMC, Xinjiang multiethnic cohort.

The total and direct effects of garlic on chronic diseases, as well as indirect effects through physical, lifestyle, and dietary factors, were investigated by SEM. The summarized total effects of garlic consumption from the three cohorts showed that dietary garlic decreased the risk of chronic diseases (Figure 1). In the XMC and UKBB cohort, garlic demonstrated a protective total effect on cancer, with effect values of −0.033 and −0.014, respectively (Supporting Information S2: Table 3). In the CLHLS and UKBB cohorts, there was a protective total effect of garlic on diabetes and hypertension, with effect values of −0.014 and −0.032 for diabetes, and −0.013 and −0.041 for hypertension, respectively (Supporting Information S2: Tables 4 and 5). Furthermore, in the UKBB cohort, a protective total effect of garlic on respiratory diseases and CVD was observed, with corresponding effect values of −0.014 and −0.035, respectively (Supporting Information S2: Tables 6 and 7). Detailed information regarding the direct and indirect pathways from garlic consumption to chronic diseases is shown in Supporting Information S2: Tables 3–7 and Supporting Information S1: Figures 2–4.

## 4 Discussion

In three large‐scale cohorts, we investigated three dietary patterns of garlic (i.e., frequency of garlic consumption, garlic intake, and preference for garlic) on the risk of the five common chronic diseases. We found a protective effect of high garlic consumption on reducing the risk of chronic diseases (Figure 2).

Previous studies have primarily investigated the associations between garlic and cancer and circulatory diseases. A large prospective cohort study in the USA reported that moderate dietary garlic consumption was associated with a reduced risk of colorectal cancer [9]. A higher intake of allium vegetables was reported to be associated with a reduced risk of cancer [27, 28]. A longitudinal follow‐up study in Iran showed that habitual intake of allium vegetables was associated with a decreased risk of CVD, hypertension, and chronic kidney disease, but did not show a significant association with the risk of diabetes [10]. However, several systematic reviews reported inconsistent results in cancer [29, 30], suggesting that the study design and population characteristics may have affected the observed outcomes.

The population‐level associations between garlic and diabetes and respiratory diseases remain unclear. In this study, we addressed this knowledge gap and found that there was a beneficial relationship between a higher garlic consumption and a lower risk of diabetes and respiratory diseases. Previous studies have typically been limited by small sample sizes from single regions or populations, and primarily focused on the association between the frequency of consuming garlic or garlic intake and individual chronic diseases [10, 27, 28]. These studies failed to comprehensively assess the effect of diverse dietary patterns on multiple chronic conditions. In contrast, our study focused on the complexity and diversity of dietary behaviors in an association analysis using three independent cohorts from different regions (i.e., older Chinese, Chinese from Xinjiang, and UK populations). We systematically evaluated the effects of various garlic dietary patterns on the risks of five common chronic diseases. Our findings provide comprehensive evidence to inform individualized dietary strategies and policies for the prevention of chronic disease.

We observed strong associations between the frequency of garlic consumption or garlic intake and a reduced risk of chronic diseases in the CLHLS cohort and XMC, while the preference for garlic showed a weaker effect. These inter‐cohort differences may have been due to dietary patterns, different populations, and measurement differences, as well as genetic and environmental factors. The possible specific reasons for these differences are as follows. Dietary differences were likely to have substantially contributed to the inter‐cohort differences. The participants in the UKBB cohort predominantly followed Western dietary patterns, with the consumption of garlic primarily through powdered seasonings, while those in the CLHLS and XMC study more frequently consumed fresh raw garlic, which contains higher concentrations of bioactive compounds to confer greater protective effects. Second, regarding different population characteristics, the participants in the UKBB cohort were generally younger and healthier at baseline, whereas those in the CLHLS cohort were older with a higher risk of chronic diseases, potentially enhancing the observed protective effects. Third, measurement differences, such as variations in garlic intake assessment and disease diagnosis, could partially explain the differential associations across the cohorts. Finally, underlying genetic and environmental factors may have further modified the observed protective effects. Therefore, geographic dietary patterns, age‐related differences, and other cohort‐specific characteristics should be considered when formulating public health recommendations.

Garlic contains various bioactive substances, primarily organosulfur compounds. These compounds have diverse effects, such as anti‐inflammatory, antioxidant, antiviral, and immunomodulatory effects [31, 32, 33]. These effects are likely to be closely related to garlic's protective and preventive mechanisms against chronic diseases. The active ingredients of garlic regulate carcinogen metabolism, induce apoptosis, inhibit cell growth and proliferation, prevent angiogenesis, and inhibit invasion and migration [34]. The antidiabetic activity of garlic can be explained by its organosulfur compounds, which block liver‐induced insulin activation, enhance insulin secretion from pancreatic beta cells, and subsequently lower blood glucose concentrations [35]. Garlic reduces high blood pressure by decreasing oxidative stress and inhibiting angiotensin‐converting enzyme [36]. Moreover, garlic can reduce cholesterol, low‐density lipoprotein, and other risk factors for CVD [37]. Additionally, garlic has considerable antiviral activity and can prevent viral infections by enhancing the immune response [38].

One important strength of this study is the use of multiple cohorts with a wide population distribution and a large sample size. We included three independent cohorts from the older Chinese population, the Chinese population in Xinjiang Uygur Autonomous Region, and the UK population for cross‐validation across three garlic consumption patterns. The detailed health screening data allowed us to assess different chronic diseases. Additionally, the findings across the three cohorts were broadly consistent, providing robust evidence. After Bonferroni correction for the five diseases, the results remained significant in the CLHLS and UKBB cohorts. Additionally, we used SEM to investigate the direct, indirect, and total effects of garlic on chronic diseases, which have not been previously examined.

However, we acknowledge that there are some limitations to this study. First, we used self‐reported information on garlic consumption, the disease status, and potential covariates, which may have led to recall bias and misclassification. Second, the garlic dietary patterns (the CLHLS used frequency, the XMC used intake, and the UKBB used preference) and dietary structures (the CLHLS studied a common Chinese diet, the XMC study examined a region‐specific diet, and the UKBB study examined a European‐American diet) were different in each cohort. Additionally, the corresponding information about garlic consumption (raw, cooked, or processed) was incomplete. These differences in the garlic dietary pattern and dietary structure, as well as an unclear cooking process, may have contributed to different associations across each cohort. Third, we adjusted for physical, lifestyle, and dietary factors, but unmeasured confounders may still have been present because of the inherent limitations of observational studies. Fourth, the use of logistic regression does not account for differences in variations in the time to events, limiting the ability to analyze changes in the risk over time and potentially introducing survival bias. Fifth, the absence of time‐to‐event data may have led to reverse causality because participants who were already ill may have altered their dietary habits as part of health management, rather than garlic intake directly affecting the disease risk.

Therefore, future studies should use Cox regression analyses with complete follow‐up cohorts to further validate these findings. Additionally, subsequent studies should investigate the causal relationships between specific garlic components, dietary patterns involving garlic, and chronic diseases, adjusting for potential confounders and applying causal inference methods. Finally, additional functional studies should be conducted to examine the underlying biological mechanisms.

## 5 Conclusions

In conclusion, high garlic consumption is associated with a lower risk of chronic diseases, namely cancer, diabetes, hypertension, respiratory diseases, and CVD. Our findings highlight the importance of garlic in preventing chronic diseases, with potential public health implications that can inform prevention policies for chronic diseases.

## Author Contributions

**Mulong Du:** conceptualization (equal), funding acquisition (equal), project administration (equal), resources (equal), supervision (equal), and writing – review and editing (equal). **Xu Qian:** methodology (equal), data curation (equal), and resources (equal). **Jianghong Dai:** methodology (equal), funding acquisition (equal), data curation (equal), and resources (equal). **Xiaoyu Tai:** conceptualization (equal), data curation (equal), formal analysis (equal), project administration (equal), visualization (equal), and writing – original draft (equal). **Tao Luo:** data curation (equal), formal analysis (equal), investigation (equal), software and writing – review and editing (equal). **Keying Song:** conceptualization (equal), data curation (equal), validation (equal), visualization (equal), and writing – original draft (equal). **Hui Zhao:** data curation (equal), investigation (equal), and supervision (equal). **Silu Chen:** data curation (equal), investigation (equal), validation (equal), and project administration (equal). **Huiqin Li:** methodology (equal), data curation (equal) and resources (equal). **Min Liu:** data curation (equal) and software (equal).

## Ethics Statement

Each cohort study was approved by the relevant ethics committee. The CLHLS was approved by the biomedical ethics committee of Peking University (IRB00001052–24713074), the UKBB study was approved by the National Research Ethics Committee (17 June 2011 [RES reference 11/NW/0382]; extended on 10 May 2016 [RES reference 16/NW/0274]), and the XMC study was approved by the ethics committee of the Academy of Traditional Chinese Medicine in Xinjiang Uygur Autonomous Region (2018XE0108).

## Consent

Each cohort study obtained informed consent.

## Conflicts of Interest

The authors declare no conflicts of interest.

## Supporting information


**Supporting Figure S1. Related to Figure 1:** Flowchart of the study.
**Supporting Figure S2. Related to Figure 2:** Structural equation modeling of the relationship between garlic consumption and chronic disease in the CLHLS cohort. (A) Cancer; (B) diabetes; (C) hypertension; (D) respiratory diseases; and (E) CVD.
**Supporting Figure S3. Related to Figure 3:** Structural equation modeling of the relationship between garlic consumption and chronic disease in the XMC. (A) Cancer; (B) diabetes; (C) hypertension; (D) respiratory diseases; and (E) CVD.
**Supporting Figure S4. Related to Figure 4:** Structural equation modeling of the relationship between garlic consumption and chronic disease in the UKBB cohort. (A) Cancer; (B) diabetes; (C) hypertension; (D) respiratory disease; and (E) CVD.

**Supporting Table S1. Related to Table 1:** Diagnosis of five chronic diseases in the CLHLS, XMC and UKBB cohorts.
**Supporting Table S2. Related to Table 2:** Baseline characteristics of participants from CLHLS, XMC and UKBB cohorts.
**Supporting Table S3. Related to Table 3:** Direct, indirect, and total effects of the garlic on cancer in the CLHLS, XMC and UKBB cohort.
**Supporting Table S4. Related to Table 4:** Direct, indirect, and total effects of the garlic on diabetes in the CLHLS, XMC and UKBB cohort.
**Supporting Table S5. Related to Table 5:** Direct, indirect, and total effects of the garlic on hypertension in the CLHLS, XMC and UKBB cohort.
**Supporting Table S6. Related to Table 6:** Direct, indirect, and total effects of the garlic on respiratory diseasess in the CLHLS, XMC and UKBB cohort.
**Supporting Table S7. Related to Table 7:** Direct, indirect, and total effects of the garlic on CVD in the CLHLS, XMC and UKBB cohort.

## Data Availability

The data of the Chinese Longitudinal Healthy Longevity Survey and the UK Biobank are available from official websites at https://opendata.pku.edu.cn and https://www.ukbiobank.ac.uk, respectively. Access to the data from the Xinjiang Multiethnic Cohort Study requires contacting the corresponding author.
