# Supplementary material for: Dietary Pattern of Garlic and Risk of Chronic Diseases: Evidence From Three Large‐Scale Cohorts
Source: Health Care Sci. 2025 Aug 3;4(4):269–80. doi: 10.1002/hcs2.70030 (PMC12371721; doi:10.1002/hcs2.70030)
Supplement: Supplementary file 1 — Supporting Figure S1. Related to Figure 1: Flowchart of the study. Supporting Figure S2. Related to Figure 2: Structural equation modeling of the relationship between garlic consumption and chronic disease in the CLHLS cohort. (A) Cancer; (B) diabetes; (C) hypertension; (D) respiratory diseases; and (E) CVD. Supporting Figure S3. Related to Figure 3: Structural equation modeling of the relationship between garlic consumption and chronic disease in the XMC. (A) Cancer; (B) diabetes; (C) hypertension; (D) respiratory diseases; and (E) CVD. Supporting Figure S4. Related to Figure 4: Structural equation modeling of the relationship between garlic consumption and chronic disease in the UKBB cohort. (A) Cancer; (B) diabetes; (C) hypertension; (D) respiratory disease; and (E) CVD. [file HCS2-4-269-s001.docx]

**SUPPLEMENTARY METHODS**

**Covariates**

The covariates analyzed in this study were as follows: age (years), sex (male or female), ethnic (CLHLS and XMC: Han Chinese or other, UKBB: white only), smoking status (yes or no), drinking status (yes or no), self-reported health (good, fair, poor), body mass index (BMI) (<18.5 kg/m², 18.5-23.9 kg/ m², 18.5-23.9 kg/m², 24-27.9 kg/m², >=28 kg/m²), dietary pattern (fish eater, meat eater, fish and poultry eater, or vegetarian).

In CLHLS, exercise was categorized as yes or no, based on whether individuals exercised regularly or not. In the XMC cohort, exercise included rarely or never, occasionally, and almost every day according to the frequency of exercise. While in UKBB, they were grouped as <150 min/week or ≥150 min/week depending on total number of minutes engaged in moderate physical activity per week. Finally, for dietary factors, in the CLHLS and XMC cohorts, fruit, vegetable, meat, fish, egg, bean, sweets, tea, and salt-preserved vegetables were categorized as rarely or never, occasionally, and almost every day based on their intake frequency. In the UKBB study, we utilized a touch-screen questionnaire to collect dietary information on fruit, vegetable, red meat, processed meat, fish, and tea intake, obtaining of fruit (pieces/day), vegetable (tablespoons/day), fish (servings/week), tea (cups/day), red meat (<2 servings/week or ≥2 servings/week), and processed meat (<2 servings/week or ≥2 servings/week). However, for egg, bean, sweets, and salt-preserved vegetables, intake information was not available. We obtained the dislike (scores 1-4), neither like nor dislike (score 5), or like (scores 6-9) information for egg, bean, sweets, salt-preserved vegetables based on the characteristics of food preferences collected from the online questionnaire as an alternative method.

**Supplementary Figure 1.** Flowchart of the study.


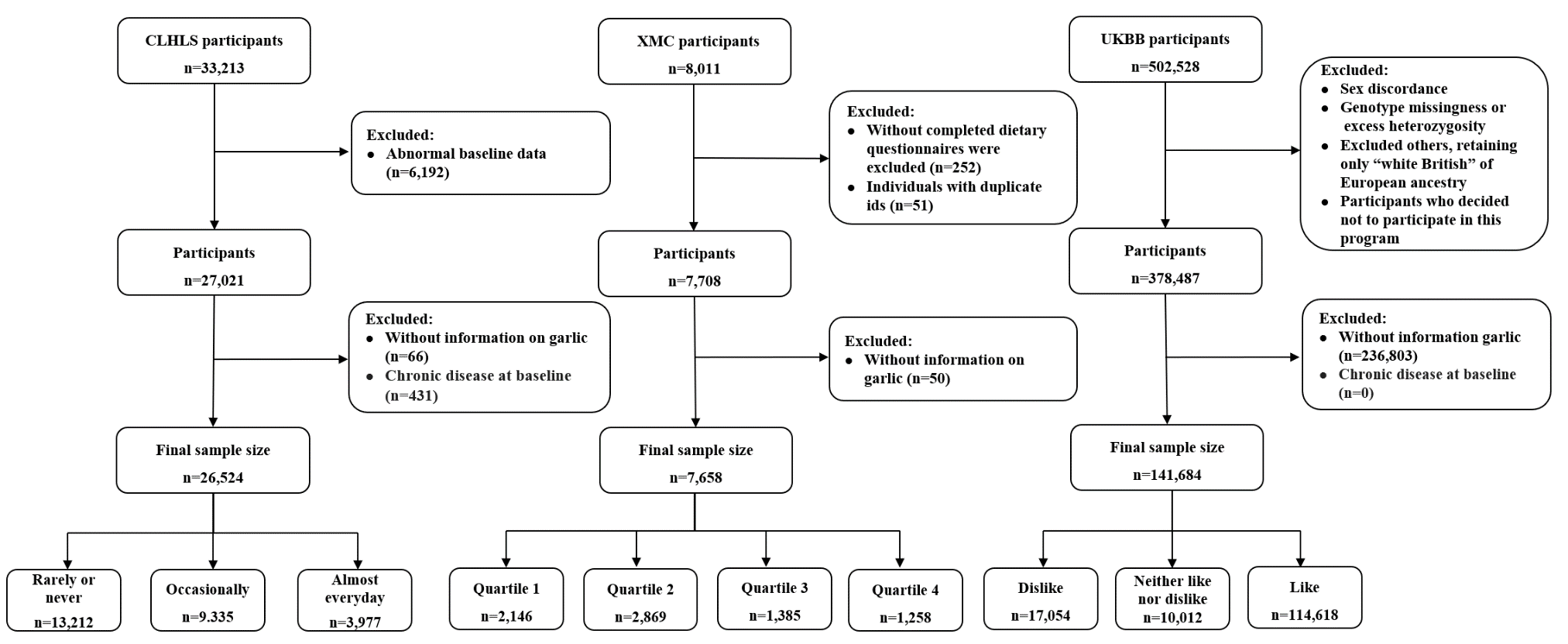


Abbreviations: CLHLS: Chinese Longitudinal Healthy Longevity Survey; XMC: Xinjiang Multiethnic Cohort Study; UKBB: UK Biobank.

**Supplementary Figure 2.** Structural equation modeling of the relationship between garlic consumption and chronic disease in the CLHLS cohort.

1. Cancer; (B) Diabetes; (C) Hypertension; (D) Respiratory diseases; (E) CVD


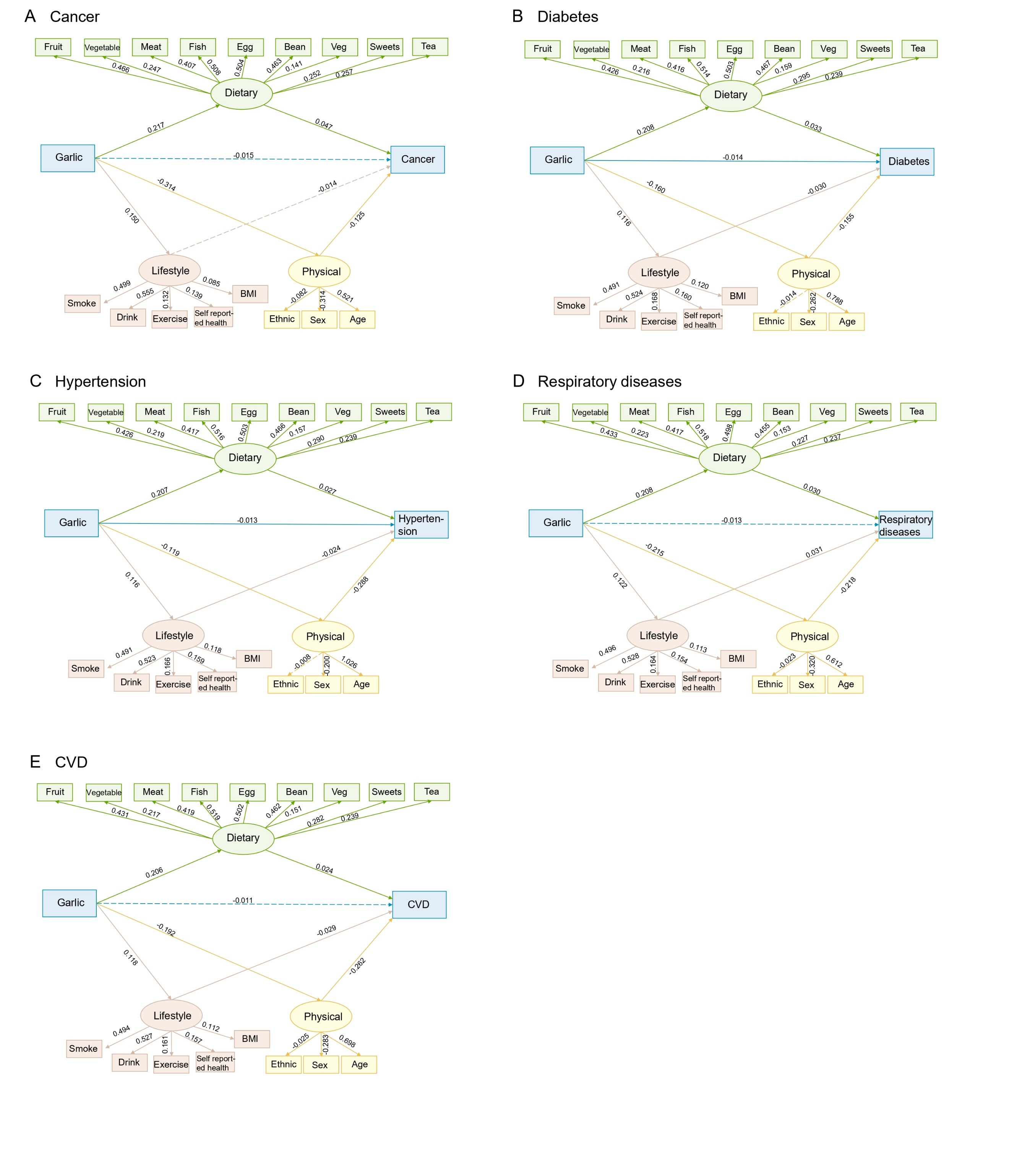


Solid lines represent p≤0.05, dashed lines represent p>0.05.

Abbreviations: CLHLS, Chinese Longitudinal Healthy Longevity Survey; CVD: cardiovascular diseases; Veg: Salt-preserved vegetables.

**Supplementary Figure 3.** Structural equation modeling of the relationship between garlic consumption and chronic disease in the XMC cohort.

1. Cancer; (B) Diabetes; (C) Hypertension; (D) Respiratory diseases; (E) CVD


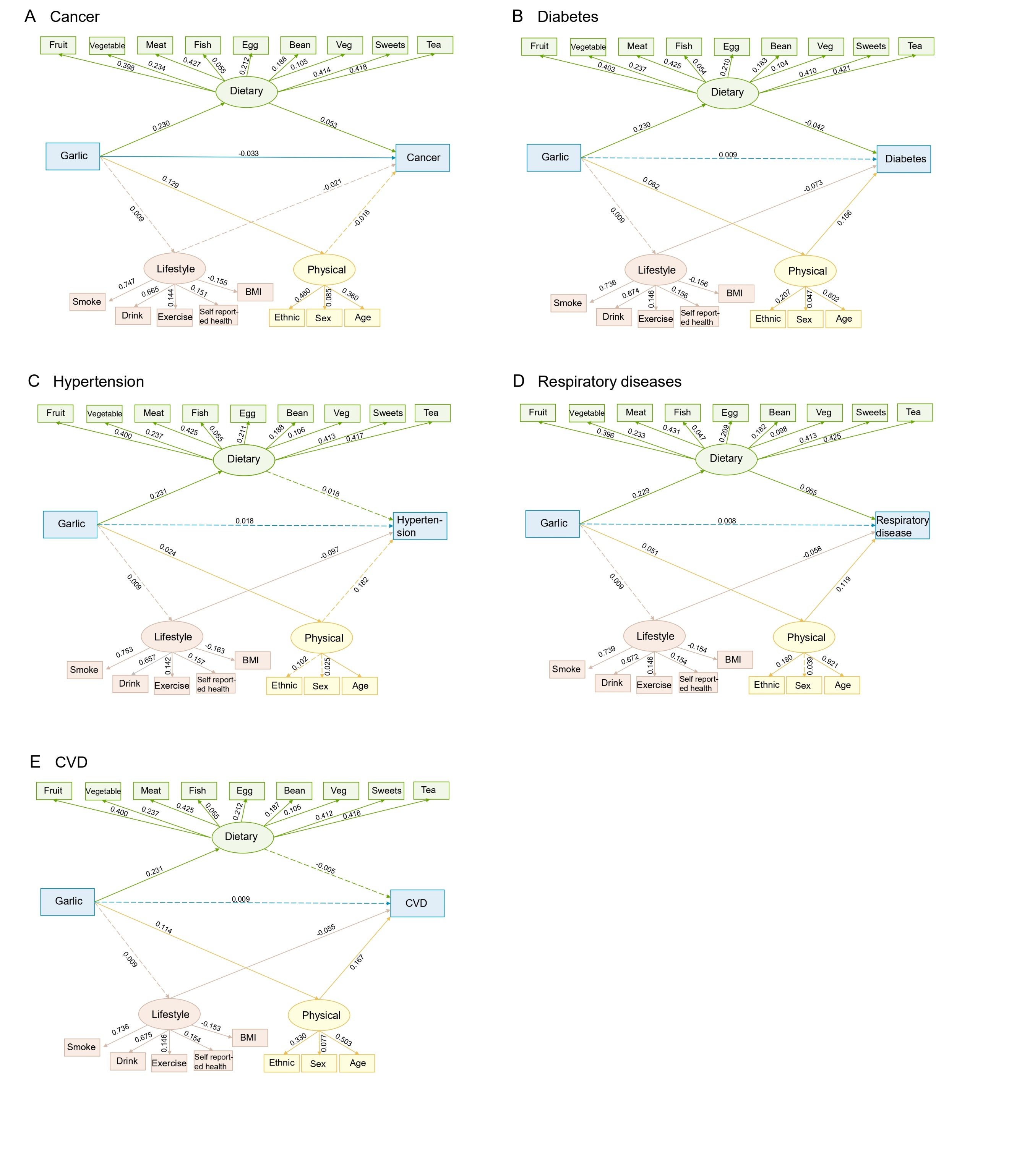


Solid lines represent p≤0.05, dashed lines represent p>0.05.

Abbreviations: XMC: Xinjiang Multiethnic Cohort Study; CVD: cardiovascular diseases; Veg: Salt-preserved vegetables.

**Supplementary Figure 4.** Structural equation modeling of the relationship between garlic consumption and chronic disease in the UKBB cohort.

1. Cancer; (B) Diabetes; (C) Hypertension; (D) Respiratory diseases; (E) CVD


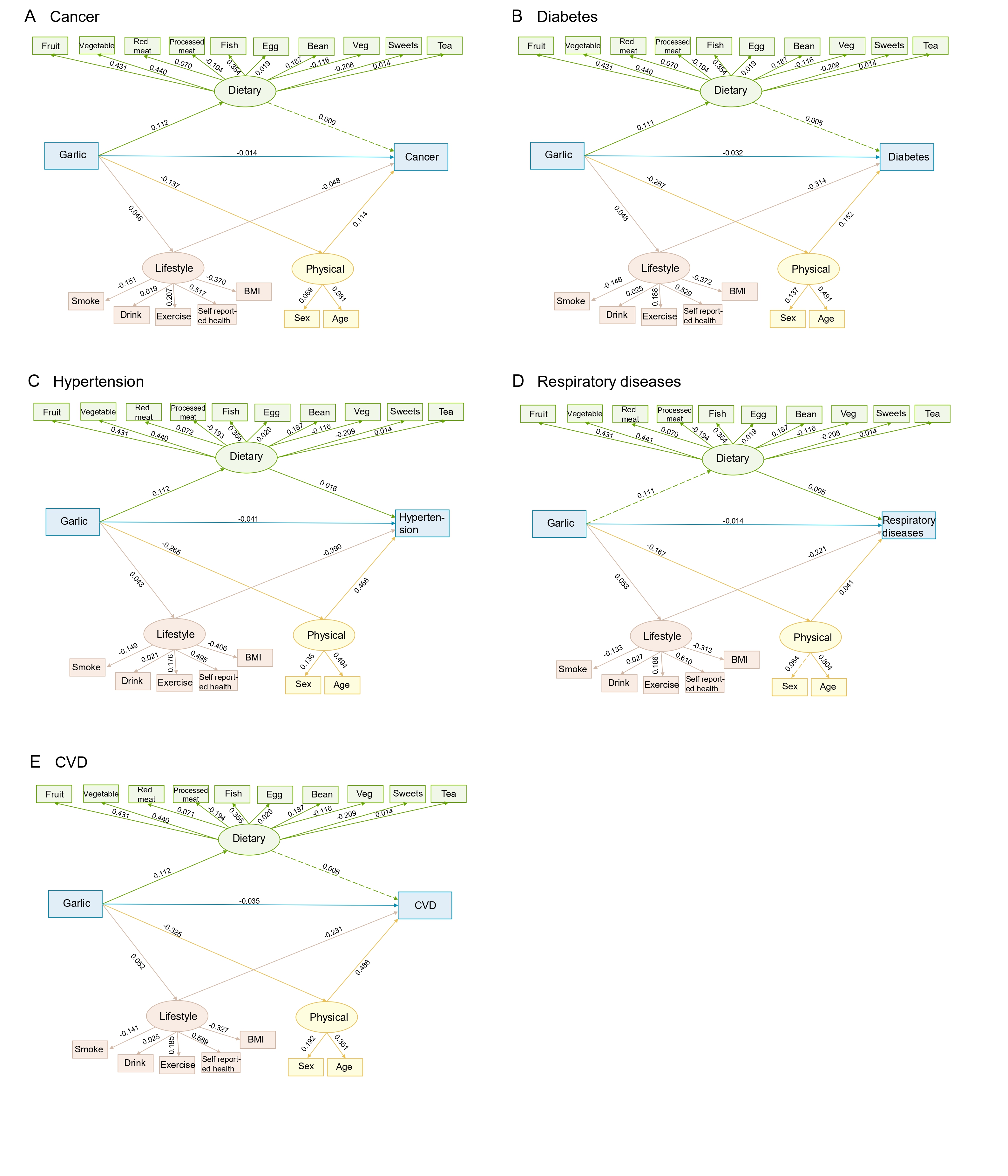


Solid lines represent p≤0.05, dashed lines represent p>0.05.

Abbreviations: UKBB: UK Biobank; CVD: cardiovascular diseases; Veg: Salt-preserved vegetables.
